# *QuickStats:* Age-Adjusted Percentage* of Adults Aged ≥18 Years Who Received an Influenza Vaccination During the Past 12 Months,^†^ by Sex and Race and Ethnicity^§^ — National Health Interview Survey, United States, 2022

**DOI:** 10.15585/mmwr.mm7248a5

**Published:** 2023-12-01

**Figure:**
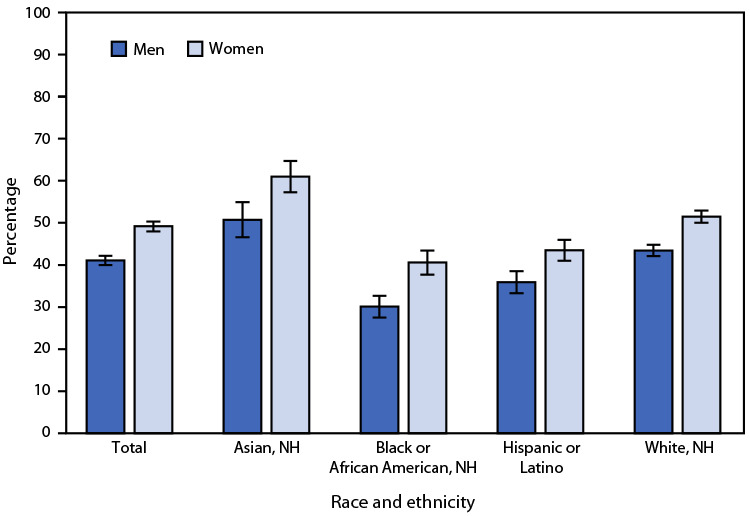
In 2022, among persons aged ≥18 years, women were more likely than were men (49.2% versus 41.1%) to have received an influenza vaccination during the past 12 months. Women were more likely than were men to have received an influenza vaccination among Asian (61.0% versus 50.7%), Black (40.6% versus 30.1%), Hispanic (43.5% versus 35.9%), and White (51.5% versus 43.4%) adults. Among men, Black adults were the least likely to have received an influenza vaccination during the past 12 months compared with Asian, Hispanic, and White adults. Among women, Black and Hispanic adults were less likely to have received an influenza vaccination during the past 12 months than were Asian and White adults.

**Source:** National Center for Health Statistics, National Health Interview Survey, 2022. https://www.cdc.gov/nchs/nhis.htm

For more information on this topic, CDC recommends the following link:  https://www.cdc.gov/flu/

